# Enhanced Adsorption Selectivity of Carbon Dioxide and Ethane on Porous Metal–Organic Framework Functionalized by a Sulfur-Rich Heterocycle

**DOI:** 10.3390/nano12234281

**Published:** 2022-12-01

**Authors:** Vadim A. Dubskikh, Konstantin A. Kovalenko, Anton S. Nizovtsev, Anna A. Lysova, Denis G. Samsonenko, Danil N. Dybtsev, Vladimir P. Fedin

**Affiliations:** 1 Nikolaev Institute of Inorganic Chemistry, Siberian Branch of Russian Academy of Sciences, 3 Acad. Lavrentiev Ave., Novosibirsk 630090, Russia; 2 Novosibirsk State University, 2 Pirogov Street, Novosibirsk 630090, Russia

**Keywords:** metal–organic framework, thieno[3,2-b]thiophenedicarboxylic acid, gas adsorption, adsorption selectivity, benzene and cyclohexane separation

## Abstract

Porous metal–organic framework [Zn_2_(ttdc)_2_(bpy)] (**1**) based on thieno [3,2-b]thiophenedicarboxylate (ttdc) was synthesized and characterized. The structure contains intersected zig-zag channels with an average aperture of 4 × 6 Å and a 49% (*v/v*) guest-accessible pore volume. Gas adsorption studies confirmed the microporous nature of **1** with a specific surface area (BET model) of 952 m^2^·g^–1^ and a pore volume of 0.37 cm^3^·g^–1^. Extensive CO_2_, N_2_, O_2_, CO, CH_4_, C_2_H_2_, C_2_H_4_ and C_2_H_6_ gas adsorption experiments at 273 K and 298 K were carried out, which revealed the great adsorption selectivity of C_2_H_6_ over CH_4_ (IAST selectivity factor 14.8 at 298 K). The sulfur-rich ligands and double framework interpenetration in **1** result in a dense decoration of the inner surface by thiophene heterocyclic moieties, which are known to be effective secondary adsorption sites for carbon dioxide. As a result, remarkable CO_2_ adsorption selectivities were obtained for CO_2_/CH_4_ (11.7) and CO_2_/N_2_ (27.2 for CO_2_:N_2_ = 1:1, 56.4 for CO_2_:N_2_ = 15:85 gas mixtures). The computational DFT calculations revealed the decisive role of the sulfur-containing heterocycle moieties in the adsorption of CO_2_ and C_2_H_6_. High CO_2_ adsorption selectivity values and a relatively low isosteric heat of CO_2_ adsorption (31.4 kJ·mol^–1^) make the porous material **1** a promising candidate for practical separation of biogas as well as for CO_2_ sequestration from flue gas or natural gas.

## 1. Introduction

Carbon dioxide is a major component of the anthropogenic pollution of the atmosphere, leading to global climate change. Extensive development of modern digital technologies, digitalization of daily life and electrification of vehicles require a continuous growth in the production of electricity, a significant part of which is generated by highly polluting coal power plants, even in developed countries [1]. Until a global transition to renewable energy, fossil fuels will need to be used for power production, that is why the decontamination of industrial flue gases from CO_2_ is an important measure to reduce irreversible damage to the environment. Adsorption technologies afford efficient solutions for separation of gases, such as CO_2_ and N_2_ (the main flue gas components), CO_2_ and CH_4_ (the main components of biogas), as well as many other industrially relevant mixtures [2]. Metal–organic frameworks (MOFs) are versatile porous materials with great adsorption characteristics [3,4,5]. Significant progress has been achieved in CO_2_ sequestration, particularly when strong Lewis acidic (uncoordinated metal cites) or Lewis basic centers (amine groups) are introduced into the porous structures [6,7,8]. Despite the obvious advantages, such porous materials usually suffer from high energy costs that are required for their reactivation. Recently, several groups have demonstrated that functionalization of porous MOFs with thiophene or selenophene heterocycles remarkably improves the selective adsorption of CO_2_ due to induced dipole interactions in the heteroatoms [9,10,11,12,13]. In particular, microporous MOFs assembled with 2,5-thiophenedicarboxylate linkers [Zn_2_(tdc)_2_(dabco)] (dabco = 1,4-diazabicyclo[2.2.2]octane) featured up to 50% greater CO_2_ adsorption uptake and adsorption selectivity compared with structurally similar [Zn_2_(bdc)_2_(dabco)], prepared using terephthalate linkers and containing no heteroatoms [9]. Importantly, such an improvement in the adsorption characteristics is not accompanied by an increase in the CO_2_ adsorption heat; thus, recycling of the porous materials could be done with minimal costs. Other than CO_2_, the selective separation of complex mixtures of hydrocarbons in natural gas and crude oil, as well as the separation of alkanes and olefines, are also very important problems for the chemical industry [14]. A number of MOFs, including those with thiophenedicarboxylate linkers, demonstrate a remarkable separation efficiency for ethane–ethylene or cyclohexane–benzene mixtures, which demonstrates the importance of further development in this direction [15,16,17,18,19,20,21]. By exploring the strategy of MOF functionalization by heterocycles, we prepared a new MOF based on the thieno[3,2-b]thiophenedicarboxylate (ttdc^2–^) organic linker which bears two thiophene moieties in its core. The adsorption of the porous material towards CO_2_ and various hydrocarbons was investigated both experimentally and theoretically. The enrichment of the microporous surface with polarizable sulfur atoms was indeed rewarded by remarkable results. In particular, quite impressive CO_2_ adsorption selectivities were obtained for CO_2_/CH_4_ and CO_2_/N_2_ gas mixtures, while the heat of CO_2_ adsorption for the new MOF was maintained at an appreciably low level. The computational DFT calculations confirm the decisive role of the sulfur-rich heterocycles in the adsorption of CO_2_ and C_2_H_6_. This study not only reports a new MOF with great adsorption properties, but validates and generalizes the approach of framework functionalization, yielding prominent porous materials suitable for the most challenging practical tasks in the separation of complex mixtures.

## 2. Materials and Methods

### 2.1. Instruments and Methods

Infrared spectra of solid samples as KBr pellets were recorded using an IR-Fourier spectrometer Scimitar FTS 2000 (4000–400 cm^−1^). The effective spectral resolution was 1 cm^−1^. The elemental analyses were obtained using an analyzer «Vario Micro-Cube». The thermogravimetric analyses were carried out in an Ar atmosphere using a NETZSCH TG 209 F1 thermoanalyzer with a heating rate of 10 deg·min^–1^ in the temperature range from 298 K to 873 K. The powder X-ray diffraction data were obtained on a «Shimadzu XRD 7000S» powder diffractometer (Cu-Kα irradiation, λ = 1.54178 Å) in the 2θ range from 5° to 30°. The porous structure was analyzed using the nitrogen adsorption technique on Quantochrome’s Autosorb iQ gas sorption analyzer at 77 K. The preliminary activation of **1** was done in the following way. The required amount of the MOF was immersed in 10 mL of acetone for 5 days. Each day the supernatant was decanted, and a new portion of acetone was added to the crystals. Then, the crystals were separated by decantation of the supernatant and dried under vacuum. The next step of activation was performed in a dynamic vacuum at 453 K for 6 h directly in the gas sorption analyzer. The nitrogen adsorption–desorption isotherms were measured within the range of relative pressures from 10^−6^ to 0.995. The specific surface area was calculated from the data obtained using the conventional BET, Langmuir and DFT models. Gas (CO_2_, CH_4_, N_2_, O_2_, CO, C_2_H_2_, C_2_H_4_ and C_2_H_6_) adsorption isotherm measurements at 273 and 298 K were carried out volumetrically on Quantochrome’s Autosorb iQ equipped with a thermostat TERMEX Cryo-VT-12 to adjust the temperature with 0.1 K accuracy. Adsorption–desorption isotherms were measured within the range of pressures from 1 to 800 torr. The database of the National Institute of Standards and Technology [22] was used as a source of *p*–*V*–*T* relations at experimental pressures and temperatures.

### 2.2. X-ray Crystallography

Diffraction data for single-crystal **1** were obtained at 150 K on a Bruker D8 Venture diffractometer equipped with a CMOS PHOTON III detector and IμS 3.0 source (λ(MoKα) = 0.71073 Å, φ- and ω-scans). Absorption corrections were applied using SADABS [23]. The structures were solved by a dual space algorithm (SHELXT [24]) and refined by the full-matrix least squares technique (SHELXL [25]) in the anisotropic approximation (except hydrogen atoms). Positions of hydrogen atoms in organic ligands were calculated geometrically and refined in the riding model. The crystallographic data and details of the structure refinements are summarized in Table S1. The structure contains a large void volume occupied with highly disordered DMA guest molecules, which could not be refined as a set of discrete atomic positions. The final composition of compound **1** was defined according to the PLATON/SQUEEZE procedure [26] (251 *e*^−^ in 737 Å^3^) and the data from element (C, H, N, S) analyses. CCDC 2212135 contains the supplementary crystallographic data for this paper. These data can be obtained free of charge from The Cambridge Crystallographic Data Center at https://www.ccdc.cam.ac.uk/structures/. The crystal data and structure refinement for **1** is also shown in Table S1.

### 2.3. Synthesis of [Zn_2_(ttdc)_2_(bpy)]·3DMA (1)

Zinc (II) nitrate hexahydrate (59.4 mg, 0.2 mmol), thieno[3,2-b]thiophene-2,5-dicarboxylic acid (H_2_ttdc, 45.6 mg, 0.2 mmol), 4,4′-bipyridine (bpy, 15.6 mg, 0.1 mmol) and 5 mL of N,N-dimethylacetamide (DMA) were placed in a glass vial with a screw cap. The reaction mixture was sonicated for 30 min and then heated at 383 K for 2 days. The resulting crystals were washed with DMA (3 × 5 mL) and dried in air. Yield 70.5 mg (70%). Anal. Calc. for [Zn_2_(ttdc)_2_(bpy)]·3DMA·H_2_O = C_38_H_41_N_5_O_12_S_4_Zn_2_ (%): C 44.8, H 4.0, N 6.7, S 12.6%. Found: C 44.7, H 3.8, N 6.7, S 12.7%. IR data (cm^−1^): 420 (w), 499 (w), 568 (w), 593 (w), 641 (m), 688 (w), 729 (w), 770 (m), 811 (m), 853 (w), 898 (w), 1016 (w), 1047 (w), 1071 (m), 1098 (w), 1191 (w), 1216 (w), 1265 (w), 1328 (s), 1381 (s), 1414 (s), 1485 (s), 1577 (s), 1611 (s), 2922 (w), 3093 (w), 3415 (w, broad).

### 2.4. Liquid Phase Separation Experiments

As-synthesized **1** (0.100 g) was placed in a closed vial containing 10 mL of a 1:1 (*v/v*) benzene–cyclohexane mixture for 5 days. Then, the crystals were very quickly filtered, washed with two 5 mL portions of methanol and transferred into a vial where 0.7 mL of d_6_-dimethyl sulfoxide (DMSO-d_6_) and several drops of concentrated HCl were added. The system was sonicated in an ultrasound bath for 10 min. The solution was transferred into a 5 mm NMR tube and an ^1^H NMR spectrum of a mixture was recorded. The ratio of benzene and cyclohexane in the mixture was determined from the ratio of the integrals of the peaks corresponding to benzene (7.3–7.4 ppm) and cyclohexane (1.4 ppm), taking into account the number of protons.

### 2.5. Computational Details

All density functional theory (DFT) calculations were carried out under periodic boundary conditions with the Vienna Ab initio Simulation Package (VASP 5.4.4) [27] using the projector-augmented wave (PAW) method [28]. We employed PBE PAW potentials (v. 5.4) for all elements and exchange–correlation functionals. The Zn, S, O, N and C atoms, and the 3d4p, 3s3p, 2s2p, 2s2p and 2s2p electrons were considered as valence electrons, while for the H atoms, the 1s electron was explicitly treated.

Structural relaxations were performed using the PBE functional [29] with D3(BJ) dispersion correction [30,31] and a plane-wave kinetic energy cutoff of 400 eV without any geometrical constraints. The final energies of species under study were calculated by the recently developed SCAN+*r*VV10 method [32], which shows outstanding performance in molecular adsorption studies, in combination with a plane-wave kinetic energy cutoff of 500 eV on top of PBE-D3(BJ) structures.

The electronic (ionic) convergence criterion was set to 10^–6^ (10^–5^) eV and Gaussian smearing with a smearing width of 0.05 eV was used throughout the computations. Only the Γ point was used for sampling the Brillouin zone during structural relaxations, whereas for single-point calculations the Brillouin zone was integrated using Γ-centered grids with a consistent spacing density employing a KSPACING parameter set to 0.5 Å^–1^.

The difference between the energy of adsorbed system (*E*_guest_@_host_) and the sum of energies of the empty metal–organic framework (*E*_host_) and non-coordinated guest molecule (*E*_guest_) was used to compute the adsorption energies (Δ*E*) according to expression:

Δ*E* = *E*_guest_@_host_ − (*E*_guest_ + *E*_host_).

Hirshfeld partial charges [33] were calculated at the PBE-MBD@rsSCS level of theory [34,35,36] using the same settings as for the SCAN+*r*VV10 single-point calculations.

To find preferential configurations of CO_2_ and C_2_H_6_ molecules inside the [Zn_2_(ttdc)_2_(bpy)] porous framework, a multiscale computational technique was used. In the first step, the noncovalent interaction (NCI)/iMTD algorithm in conformer–rotamer ensemble sampling tool (CREST) [37,38] coupled with force-field GFN-FF [39] and extended tight binding GFN2-xTB [40] methods (GFN2-xTB//GFN-FF approach; GFN-FF and GFN2-xTB calculations were performed with the xTB [41] package) were applied to a model system consisting of one guest molecule and a representative finite cluster, which was cut from the experimental [Zn_2_(ttdc)_2_(bpy)] structure. In the second step, a number of the most relevant structures obtained in the previous computational step were recomputed using periodic DFT calculations with a subsequent stability check using simulated annealing.

## 3. Results and Discussion

**Scheme 1:**
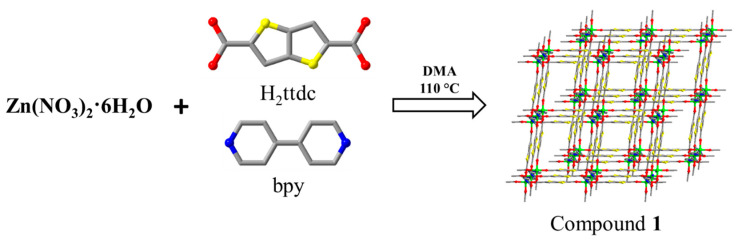
Scheme of the synthesis of **1**.

Single crystals of the studied compound [Zn_2_(ttdc)_2_(bpy)]·3DMA (**1**) were isolated in a high yield in a solvothermal reaction of Zn(II) nitrate, H_2_ttdc and bpy (2:2:1 molar ratio) in N,N-dimethylacetamide (DMA) at 110 °C (Scheme 1). The phase purity of the sample was confirmed by powder X-ray diffraction (Figure S1), its chemical composition was established by elemental analysis and additionally confirmed by FT-IR (Figure S2).

**Figure 1:**
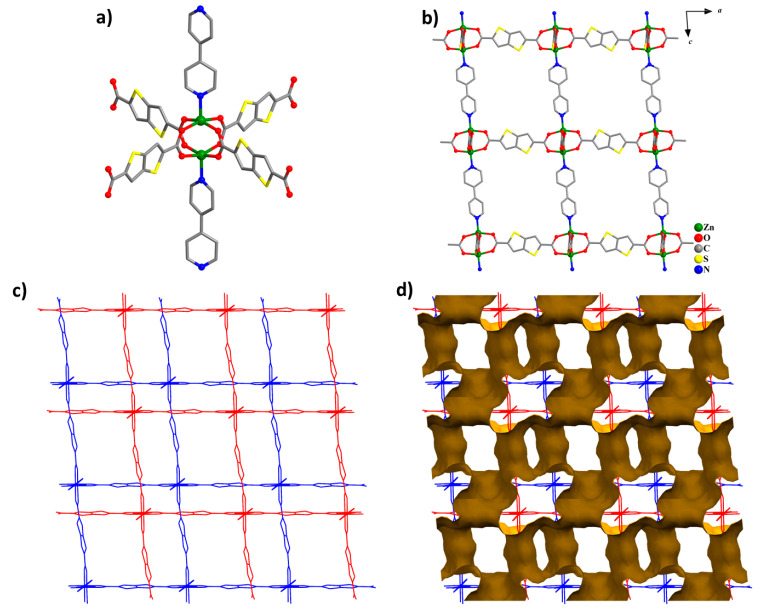
**(a**) {Zn_2_(COO)_4_N_2_} “paddle-wheel” complex in 1; (**b**) the framework structure of **1** in the *ac* plane; (**c**) two interpenetrated networks in **1** in the *ab* plane; (**d**) pore structure of **1** in the *ab* plane.

The compound **1** is based on a binuclear tetracarboxylate “paddle-wheel” complex {Zn_2_(COO)_4_N_2_}, where each Zn(II) cation adopts a square-pyramidal geometry of four O atoms of carboxylate groups, capped with the N atom of the bpy ligand (Figure 1a). Such complexes act as 6-connected building blocks with octahedron geometry. Every block is linked to four others blocks by the ttdc^2–^ ligands, located around the equator of the octahedron, and to two blocks by the bpy ligands in the apical positions of the octahedron (Figure 1b). The overall connectivity of the metal–organic network in **1** is primitive cubic (**pcu**). The overall crystal structure of **1** is composed of two identical interpenetrated pcu nets related to each other via the inversion center (Figure 1c). Interestingly, there is a similar coordination compound [Zn_2_(ndc)_2_(bpy)], synthesized with a 2,6-naphthalenedicarboxylate linker [42,43], whose size and geometry are almost the same as for ttdc^2–^. While the activated compound [Zn_2_(ndc)_2_(bpy)] possesses dense, triply interpenetrated **pcu** nets with almost no guest-accessible open space, the double network interpenetration in **1** results in microporous zig-zag channels with an average aperture of 4 × 6 Å, intersected with smaller windows of ca. 4 Å in diameter. The internal surface of the microporous structure is lined with aromatic groups of the bpy ligands and thienothiophene moieties. The as-synthesized compound **1** contains solvent DMA molecules determined from the X-ray diffraction data and confirmed by other analyses. Assuming a solvent-free framework, the calculated guest-accessible volume of **1** is 49% (*v/v*); a rather substantial value taking the interpenetration into account. The pore structure of **1** is shown in Figure 1d.

While the interpenetration inevitably reduces the volume of the pores, it also improves the general stability and robustness of the framework as well as the gas adsorption at lower partial pressures. Being encouraged by the heterocycle-rich surface of the potentially porous structure [Zn_2_(ttdc)_2_(bpy)], we carried out an extensive investigation of the gas adsorption properties of the compound. Thermogravimetric analysis indicates that guest molecules evaporate from the pores up to 200 °C while an irreversible decomposition of the metal–organic framework takes place above 250 °C (Figure S3).

### 3.1. Adsorption Studies

Liquid phase adsorption experiments were carried out by immersing the crystals of **1** in a 1:1 (by volume) mixture of benzene and cyclohexane. The crystals were collected, rinsed and digested in DMSO with some amount of HCl. The composition of the organic guest molecules in the final solution was analyzed by ^1^H NMR, which reveals a C_6_H_6_:C_6_H_12_ = 14:1 relative molar ratio (Figure S4). The pronounced affinity of **1** to the aromatic benzene over the aliphatic cyclohexane could be expected considering the exclusively aromatic nature of the environment of the micropores formed by bpy and ttdc^2–^ ligands. Nevertheless, the obtained high selectivity value (C_6_H_6_:C_6_H_12_ = 14:1) exceeds many of the literature data acquired under the same conditions as there are just a few porous MOFs with better or comparable performance [16,44,45,46,47]. The obtained results demonstrate promising perspectives of **1** in purification of the cyclohexane from benzene, which is a necessary step in the synthesis of caprolactam and production of important polyamide polymers and fibers, such as Nylon.

**Figure 2:**
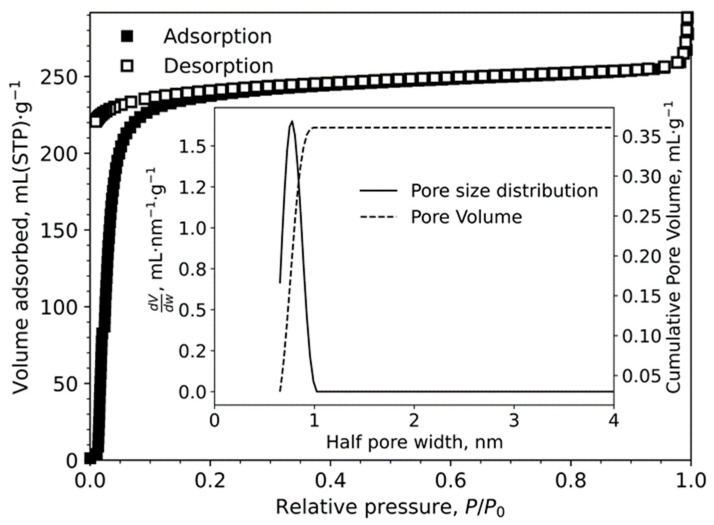
Nitrogen adsorption–desorption isotherms at 77 K and pore volume distribution (insert).

**Figure 3:**
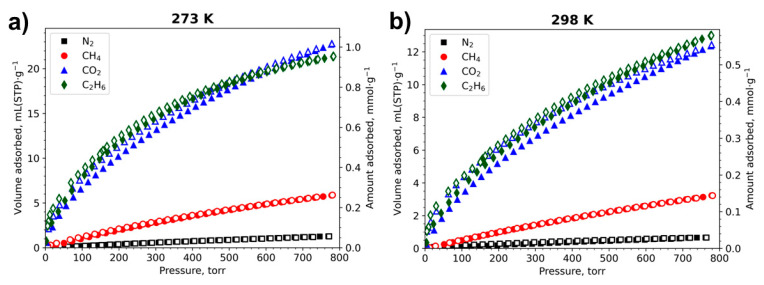
Adsorption and desorption isotherms of N_2_, CH_4_, CO_2_ and C_2_H_6_ at 273 K (**a**) and 298 K (**b**) for **1a**.

The activation of **1** was successfully carried out by DMA substitution by acetone, followed by a vacuum treatment at 180 °C for 6 h. The N_2_ adsorption data of the activated [Zn_2_(ttdc)_2_(bpy)] (**1a**) at *T* = 77 K reveal a typical type I reversible adsorption isotherm, characteristic of microporous materials (Figure 2), confirming the permanent porosity of the sample. The obtained pore volume *V*_pore_ = 0.37 cm^3^·g^–1^ (DFT, Table S2) coincides with the expected porosity (0.36 cm^3^·g^–1^) calculated from the guest-free volume and the crystal density of **1a**, which proves the completeness of the activation and stability of the porous MOF. The specific surface area (BET model) is 952 m^2^·g^–1^ (Table S2). A pore size distribution plot (DFT method) shows the presence of narrow pores with a diameter of less than 1 nm, which is in an agreement with single-crystal X-ray diffraction data. The results of the adsorption of small “inorganic” gases (CO, CO_2_, O_2_, N_2_) as well as “organic” light hydrocarbons (CH_4_, C_2_H_2_, C_2_H_4_, C_2_H_6_) at 273 K and 298 K are presented in Figure 3 and Figures S5 and S6 and Table 1.

Among the others, the adsorption of gases with higher boiling points, such as CO_2_ and C_2_ hydrocarbons, is the most significant. Particularly, the 1 bar CO_2_ adsorption capacity of **1a** is 22.3 cm^3^·g^–1^ (1 mmol·g^–1^) at 273 K and 12.1 cm^3^·g^–1^ (0.54 mmol·g^–1^) at 298 K. Such values are typical for other porous MOFs with no strong binding centers and comparable surface area, e.g., ZIF-71 [48]. The zero-coverage isosteric heat *Q*_st_(0) of the CO_2_ adsorption, calculated from the isotherm data, is 31.4 kJ·mol^–1^ (Table S4), supporting the absence of specific adsorption centers, such as open metal sites or amine groups. The adsorption capacities of the ethane, ethylene and acetylene are very comparable: 20.8–22.1 cm^3^·g^–1^ (*ca*. 0.95 mmol·g^–1^) at 273 K and 12.8–14.8 cm^3^·g^–1^ (*ca*. 0.6 mmol·g^–1^) at 298 K. The experimental *Q*_st_(0) values for C_2_H_2_ (36.2 kJ·mol^–1^) and C_2_H_6_ (35.3 kJ·mol^–1^) are noticeably higher than that for C_2_H_4_ (32.1 kJ·mol^–1^) (Table S4). Stronger adsorption interactions for acetylene at low surface coverage is likely attributed to the acidic nature of the C_2_H_2_ protons and possible hydrogen-bonding with the carboxylate groups of the framework. Since no specific interaction of the framework with π-electrons of C_2_H_4_ could be realized, the somewhat stronger affinity of **1a** to ethane over ethylene could simply be explained by a greater number of hydrogen atoms and multidirectional interatomic contacts in the former.

The adsorption selectivity (*S*_ads_) for a binary gas mixture could be assessed by three commonly used approaches. The ratio of adsorption uptakes, *V*_1_/*V*_2_, at 1 bar better indicates the selectivity at ambient conditions, while a ratio of Henry’s constants relates to the selectivity at low pressure range. A more complex Ideal Adsorption Solution Theory (IAST) allows selectivity calculations at any molar ratio or pressure range. The corresponding data are summarized in Table 2. There is no meaningful selectivity between C_2_ hydrocarbons as the corresponding values are low no matter what criteria are used for the estimations. However, there are fairly good ethane adsorption selectivity values for an equimolar mixture of ethane and methane: *S*_ads_ = 12.0 at 273 K and *S*_ads_ = 14.8 at 298 K (IAST). Even better values could be expected for a C_2_H_6_/CH_4_ = 1:9 mixture, more relevant to the selective extraction of ethane from natural gas or shale gas, as the corresponding values are *S*_ads_ = 28.4 at 273 K and *S*_ads_ = 39.1 at 298 K. It should be mentioned that even a moderate adsorption selectivity of *S*_ads_ = 8 is considered to be high enough for practical separation applications [49].

The separation of mixtures of methane and carbon dioxide is relevant to a number of practical applications, such as a separation of biogas (mainly CO_2_ and CH_4_) and purification of natural gas or shale gas from the corrosive components to prevent damage to gas pipeline infrastructure. Even though a typical concentration of CO_2_ in natural gas rarely exceeds 1%, in certain gas deposits the molar content of CO_2_ can reach up to 20% and must therefore be reduced before further processing. In this regard, porous materials with an as high as possible preferable adsorption of CO_2_ over CH_4_ are required. The IAST calculated CO_2_ adsorption selectivities of **1a** for the equimolar mixture of CH_4_ and CO_2_ are *S*_ads_ = 14.4 (273 K) and *S*_ads_ = 11.7 (298 K). According to a very recent review, these values are among the top 15% of ca. 400 reported selectivity data for MOFs [50]. It must be mentioned that the majority of those top 15% of highly selective MOFs contain either strong Lewis acidic adsorption centers (unsaturated metal sites) or strong Lewis basic adsorption centers (lone electron pairs on nitrogen atoms) which improves their selective adsorption of CO_2_ due to strong chemical interactions with those centers. On the other hand, a respective amount of heat energy must be sacrificed to overcome the high adsorption enthalpy of CO_2_ to regenerate such materials in a repeating sequestration process (vide infra). The porous **1a** does not have the above disadvantages yet demonstrates a competitively high CO_2_/CH_4_ adsorption selectivity. Moreover, much greater separation efficiency could be expected for the methane-rich mixtures mimicking the typical concentration of CO_2_ in natural gas CO_2_:CH_4_ = 1:9 (*S*_ads_ = 29.1 at 298 K) and for CO_2_:CH_4_ = 1:99 (*S*_ads_ = 93.7 at 298 K), promising a strong potential of the title compound in natural gas purification applications.

In the case of the CO_2_/N_2_ gas mixtures, the IAST calculations result in rather high adsorption selectivity performance. For an equimolar mixture of CO_2_/N_2_, the adsorption selectivity factors are *S*_ads_ = 27.2 and 34.0 at 298 K and 273 K, respectively. In the case of CO_2_/N_2_ = 15:85, which is typical of the composition of flue gas in coal power plants, the adsorption selectivity factors reach even greater values, *S*_ads_ = 56.4 at 298 K and 73.6 at 273 K. Those are very high numbers, rivaling the best data reported in the literature for MOFs with no strong CO_2_ adsorption centers [51,52,53,54,55,56,57]. As mentioned already, CO_2_ molecules readily interact with unsaturated metal sites or amine groups, but an introduction of such strong adsorption centers into a MOF structure does not necessarily lead to the desired outcome. For example, well-known MOFs mmen@[Mg_2_(dobpdc)] and mmen@[Cu_3_(bttri)_2_] (dobdc = 2,5-dioxido-1,4-benzenedicarboxylate; bttri = 1,3,5-tri(1*H*-1,2,3-triazol-4-yl)benzene), functionalized by N,N′-dimethylethylenediamine (mmen) possess remarkably high CO_2_/N_2_ selectivity values of *S*_ads_ = 200 and 327, respectively [58,59]; however, substantial CO_2_ adsorption heats (71 and 96 kJ·mol^–1^, respectively) make practical utilization of such materials problematic. In this regard, a porous material with a less prominent, yet still high, CO_2_ selective adsorption and energy-friendly regeneration requirements, such as the studied compound **1a**, could be a viable compromise, suitable for a practical separation of biogas as well as for CO_2_ sequestration from flue gas or natural gas.

It is interesting to compare the CO_2_ adsorption properties of the three porous compounds [Zn_2_(bdc)_2_(dabco)], [Zn_2_(tdc)_2_(dabco)] and [Zn_2_(ttdc)_2_(bpy)] (**1a**), which are based on identical {Zn_2_(RCOO)_4_N_2_} “paddle-wheel” units and share the same primitive-cubic topology in their frameworks. Despite some differences in general structural features and composition, these structures have more or less comparable volumetric pore volumes and surface areas (Table S7). On the other hand, the chemical environment of the channels is very different and primarily depends on the carboxylate linker. The first structure, [Zn_2_(bdc)_2_(dabco)], contains terephthalate anions and therefore has no aromatic heterocycles. The second structure, [Zn_2_(tdc)_2_(dabco)], contains thiophene rings with two thiophenedicarboxylate anionic linkers per formula unit, or one sulfur atom per 517 Å^3^ of the unit cell volume. The sulfur-rich thienothiophene moieties, as well as the framework interpenetration in **1a**, apparently results in even denser lining of the heterocycles on the inner surface of **1a**, as one S atom corresponds to 276 Å^3^ volume of the unit cell. It was demonstrated earlier that the sulfur-containing heterocycle serves as a secondary CO_2_ adsorption site via induced dipole interactions [9,10]. Even though such interactions are not as strong as dipole–dipole interactions existing between a CO_2_ molecule and a polar “paddle-wheel” unit, the incorporation of thiophene was proven to increase the CO_2_ adsorption capacity and CO_2_/N_2_ adsorption selectivity for [Zn_2_(tdc)_2_(dabco)] compared with those of [Zn_2_(bdc)_2_(dabco)]. In the case of **1a**, the IAST adsorption selectivity (*S*_ads_ = 27.2), calculated under ambient conditions (298 K, 1 bar, CO_2_:N_2_ = 1:1), greatly surpasses the corresponding data for [Zn_2_(tdc)_2_(dabco)] (*S*_ads_ = 11.2) and [Zn_2_(bdc)_2_(dabco)] (*S*_ads_ = 9.2). Albeit there are a number of differences between **1a** and its prototypes [Zn_2_(tdc)_2_(dabco)]/[Zn_2_(bdc)_2_(dabco)] such as channel shape, degree of interpenetration, nature of the auxiliary N-donor linker, etc., the contribution of the sulfur-rich thiophene heterocycles to the enhanced adsorption selectivity of the porous material towards CO_2_ must not be ignored. The obtained experimental data fully confirm an earlier hypothesis regarding the feasibility of auxiliary interactions between the gas molecules and aromatic heterocycles in effective CO_2_ binding while maintaining low heat of the adsorption.

### 3.2. Theoretical Studies

**Figure 4:**
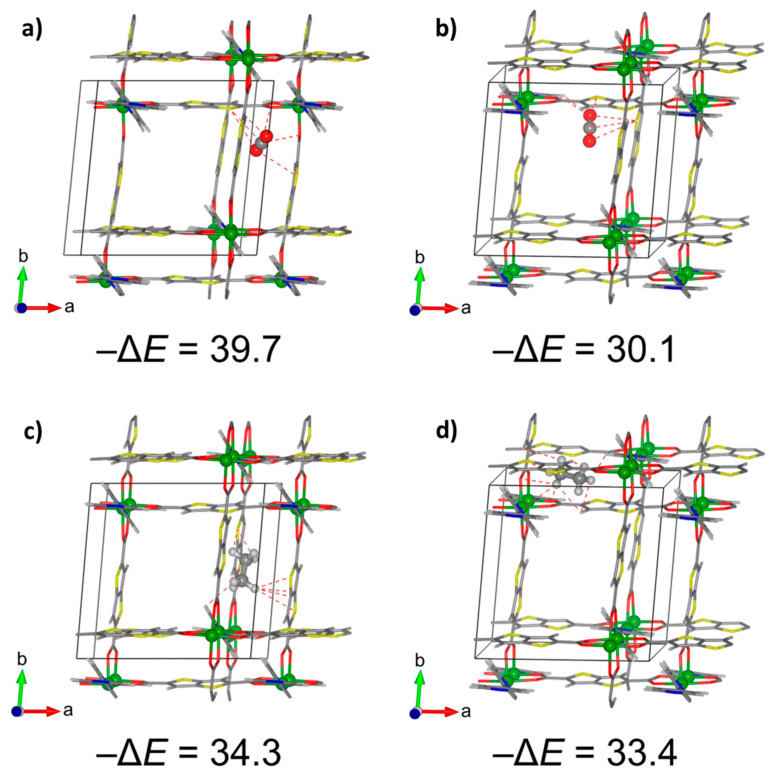
Calculated structures and adsorption energies (Δ*E*, kJ·mol^–1^) of CO_2_ (**a**,**b**) and C_2_H_6_ (**c**,**d**) molecules at the most relevant adsorption sites inside [Zn_2_(ttdc)_2_(bpy)] pores. Short host–guest interatomic contacts are indicated by red dashed lines (a single guest molecule is shown). Color code: Zn (green), S (yellow), O (red), N (blue), C (gray), H (light gray).

To probe the nature of host–guest interactions, we have computationally studied the adsorption of CO_2_ and C_2_H_6_ molecules inside the porous framework [Zn_2_(ttdc)_2_(bpy)], which has demonstrated high affinity to these gases. By using a computational approach based on the dispersion-corrected density functional theory method combined with periodic boundary conditions (SCAN+*r*VV10//PBE-D3(BJ) level of theory [29,30,31,32]), we have found the lowest energy orientations of CO_2_ and C_2_H_6_ molecules physically adsorbed on the surface of [Zn_2_(ttdc)_2_(bpy)], shown in Figure 4. It turned out that the most preferential CO_2_ adsorption centers are located near the polar carboxylate groups of the “paddle-wheel” units (Figure 4a), which is consistent with a previous study [9]. The structural analysis (Figure S12) reveals that the CO_2_ molecules occupy the symmetrical corner position establishing two C(CO_2_)···O(COO) contacts between the positively charged C atom of carbon dioxide and negatively charged O atoms of two carboxylate moieties, respectively. The rather short C···O interatomic distances (*r* = 3.02–3.16 Å) are most favorable from an electrostatics perspective, although there are also some longer intermolecular contacts between CO_2_ and organic ligands. Depending on the particular orientation of CO_2_ around the “paddle-wheel”, the binding energies (Δ*E*) of this adsorption site vary between ca. 30 and 40 kJ·mol^–1^, which is very much consistent with the experimental *Q*_st_(0) adsorption heat value (31.4 kJ·mol^–1^). On the other hand, organic linkers provide a somewhat less effective, but a numerous and structurally diverse, secondary adsorption site (Figure 4b). Depending on its particular position in the channel of **1**, the CO_2_ molecule interacts with one, two or three thienothiophene moieties and, in some cases, with the bpy linker (Figure S12). It should be pointed out that the number of the interatomic contacts of ttdc^2–^ anions with the CO_2_ molecule at those secondary adsorption sites is considerably greater than the number of such contacts with bpy (ligand stoichiometry is considered), unambiguously suggesting that sulfur-rich heterocycles establish a quite effective environment for CO_2_ adsorption. The total CO_2_ binding energies of the secondary adsorption sites are relatively low, Δ*E* ≈ 20 ÷ 30 kJ·mol^–1^ (Figure 4b and Figure S12), yet exceed the CO_2_ vaporization heat (16.7 kJ·mol^–1^), which eventually drives the adsorption. Even though the induced dipole intermolecular interactions may not be as significant as the coulomb interactions between polar atoms, the high density of the thiophene groups in the micropores of **1** (vide supra) and numerous CO_2_⋅⋅⋅ttdc^2–^ contacts ensures an apparent increase in the specific CO_2_ adsorption selectivity over non-polar gases such as N_2_ and CH_4_. Again, these results fully support the previous hypotheses that MOF functionalization by thiophene heterocycles favors CO_2_ adsorption at the same time as keeping the CO_2_ adsorption heat at a minimum, since weaker van-der-Waals interactions of the secondary adsorption sites do not contribute much to the total binding energy.

Interestingly, the opposite situation is observed in the case of ethane hydrocarbon adsorption, which prefers to bind with the organic linkers rather than with the polar “paddle-wheel” units. Such distinct adsorption behavior can be explained by the different polarity of the C–O and C–H bonds of guest molecules. Indeed, the C_2_H_6_ molecule in its adsorption centers tends to interact with less charged host atoms giving rise to several C–H···H (*r* = 2.73–2.89 Å), C–H···C (*r* = 2.90–3.06 Å) and C–H···S (*r* = 3.30–3.72 Å) contacts that are mainly stabilized by electrostatic and dispersion forces (Figure S13). It is necessary to note that the most energetically preferred CO_2_ and C_2_H_6_ positions on the framework’s surface are characterized by a number of favorable interatomic contacts between guests and sulfur atoms, highlighting the important role of sulfur-containing heterocycles in effective adsorption of such gases.

According to the calculated binding energy values, both carbon dioxide and ethane have comparable strengths of host–guest interactions, which is in line with the experimental findings. Although the CO_2_ molecule binds with the primary adsorption site more strongly than C_2_H_6_ (ΔΔ*E* = –5.4 kJ·mol^–1^; Figure 4a,c), the strength of interaction between the CO_2_ molecule and the secondary adsorption site is weaker by 3.3 kJ·mol^–1^ when compared to C_2_H_6_ (Figure 4b,d). A moderate binding energy difference between primary and secondary adsorption sites suggests that studied guest molecules can interact effectively not only with polar “paddle-wheel” units of the porous [Zn_2_(ttdc)_2_(bpy)] framework but also with its heterocyclic organic moieties. Thus, the introduction of sulfur-containing linkers into the parent MOF structure enables guest molecules to form multiple C–O···S (in case of CO_2_) or C–H···S (in case of C_2_H_6_) stabilizing contacts that lead to an enhancement in adsorption uptake and selectivity observed experimentally.

## 4. Conclusions

A new microporous metal–organic framework with intersecting channels formed by thienothiophene bridging ligands has been synthesized and characterized. The compound demonstrates a selective adsorption of benzene over cyclohexane and ethane over methane, approaching the best reported values in the literature. Most interestingly, it possesses a highly desired—but quite rare combination—of a remarkable selectivity of adsorption of CO_2_ over non-polar gases (N_2_, CH_4_) and only a moderate CO_2_ adsorption heat. The theoretical DFT calculations validated the unique role of the thiophene-like heterocycle moieties in the specific binding of CO_2_ and C_2_H_6_, which emphasizes the feasibility of functionalization of porous materials by sulfur-rich heterocycles for an improvement in the adsorption characteristics.

## Figures and Tables

**Table 1:** Gas uptakes in **1a** at 273 K and 298 K.

Gas	273 K			298 K		
	cm^3^ (STP)·g^–1^	mmol·g^–1^	wt. %	cm^3^ (STP)·g^–1^	mmol·g^–1^	wt. %
CO_2_	22.3	0.99	4.2	12.1	0.54	2.3
CH_4_	5.7	0.25	0.4	3.1	0.14	0.2
N_2_	1.3	0.06	0.2	0.7	0.03	0.1
C_2_H_2_	22.1	0.99	2.5	14.8	0.66	1.7
C_2_H_4_	20.8	0.93	2.5	13.3	0.59	1.6
C_2_H_6_	21.1	0.94	2.7	12.8	0.57	1.7
CO	2.4	0.11	0.3	1.5	0.07	0.2
O_2_	1.7	0.07	0.2	1.1	0.05	0.2

**Table 2:** Selectivity factors for **1** for separation of equimolar binary gas mixtures evaluated by different approaches.

Gas Mixtures	273 K			298 K		
	*V*_1_/*V*_2_	*K_H_*_1_/*K_H_*_2_	IAST	*V*_1_/*V*_2_	*K_H_*_1_/*K_H_*_2_	IAST
CO_2_/N_2_	17.2	93.8	34.0 (73.6 ^a^)	17.3	58.8	27.2 (56.4 ^a^)
CO_2_/CH_4_	3.9	15.8	14.4 (38.3 ^b^, 143.9 ^c^)	3.9	10.6	11.7 (29.1 ^b^, 93.7 ^c^)
C_2_H_2_/C_2_H_4_	1.06	1.48	1.12	1.11	1.27	1.12
C_2_H_6_/C_2_H_2_	0.95	1.09	2.08	0.86	1.13	3.50
C_2_H_6_/CH_4_	3.7	30.5	12.0 (28.4 ^b^)	4.1	17.6	14.8 (39.1 ^b^)

^a^ for ratio of 15:85; ^b^ for ratio of 1:9; ^c^ for ratio 1:99.

## Data Availability

CCDC 2212135 contains the supplementary crystallographic data for this paper. These data can be obtained free of charge from The Cambridge Crystallographic Data Center at https://www.ccdc.cam.ac.uk/structures/.

## Supplementary Materials

The following supporting information can be downloaded at: https://www.mdpi.com/article/10.3390/nano12234281/s1, Figure S1: Powder X-ray diffraction patterns of as-synthesized **1** (black), activated **1a** (blue) and theoretically modeled from the single-crystal X-ray diffraction data (red); Figure S2: IR spectrum of **1**; Figure S3: TG curve for **1**; Figure S4: ^1^H NMR spectrum of d_6_-dimethyl sulfoxide solution of the digested **1** after adsorption of benzene and cyclohexane from the liquid phase (1:1 *v/v*); Figure S5: Adsorption and desorption isotherms of N_2_, O_2_, CO and CO_2_ at 273 K (left) and 298 K (right) for **1a**; Figure S6: Adsorption and desorption isotherms of CH_4_, C_2_H_2_, C_2_H_4_ and C_2_H_6_ at 273 K (left) and 298 K (right) for **1a**; Figure S7: Fits of isotherms by virial equations; Figure S8: Isosteric heats of adsorption of N_2_, CH_4_, CO_2_, C_2_H_2_, C_2_H_4_ and C_2_H_6_ on **1a** calculated by virial approach as a function of amount adsorbed; Figure S9: Fits of isotherms by appropriate models; Figure S10: Prediction of adsorption equilibrium by IAST (solid lines) and dependence of selectivity factors on gas phase composition (dashed lines) for binary gas mixtures (total pressure 1 bar): (a) CO_2_/N_2_; (b) CO_2_/CH_4_; (c) C_2_H_6_/C_2_H_2_; (d) C_2_H_6_/CH_4_; Figure S11: Dependence of selectivity factors on total gas pressure for equimolar binary gas mixtures: (a) CO_2_/N_2_; (b) CO_2_/CH_4_; (c) C_2_H_6_/C_2_H_2_; (d) C_2_H_6_/CH_4_; Figure S12: Calculated interatomic distances (Å) of the shortest X_guest_···Y_host_ contacts (red dashed lines) and the corresponding Hirshfeld partial charges for CO_2_ molecule adsorbed at the most relevant sites I.A, I.B, I.C, and I.D inside [Zn_2_(ttdc)_2_(bpy)] pores. Adsorption energies (Δ*E*) are given in kJ·mol^–1^. Color code: Zn (green), S (yellow), O (red), N (blue), C (gray), H (light gray); Figure S13: Calculated interatomic distances (Å) of the shortest X_guest_···Y_host_ contacts (red dashed lines) and the corresponding Hirshfeld partial charges for C_2_H_6_ molecule adsorbed at the most relevant sites II.A and II.B inside [Zn_2_(ttdc)_2_(bpy)] pores. Adsorption energies (Δ*E*) are given in kJ·mol^–1^. Color code: Zn (green), S (yellow), O (red), N (blue), C (gray), H (light gray); Table S1: Crystal data and structure refinement for **1**; Table S2: The parameters of porous structure of **1a**; Table S3: Virial coefficients *A_i_* and *B_j_* for gas adsorption isotherms at 273 K and 298 K on 1a; Table S4: Zero coverage heats of adsorption in kJ·mol^–1^; Table S5: Henry constants for gas adsorption on 1a in mmol·g^−1^·bar^−1^ at 273 K and 298 K obtained by virial approach; Table S6: Fitted parameters for adsorption isotherms on 1a at 273 K and 298 K and corresponding Henry constants; Table S7: The parameters of porous structure of [Zn_2_(bdc)_2_(dabco)], [Zn_2_(tdc)_2_(dabco)] [2] and [Zn_2_(ttdc)_2_(bpy)] (**1a**, this work). Reference [60] is cited in the supplementary materials.
