# Supplementary material for: Enhanced Adsorption Selectivity of Carbon Dioxide and Ethane on Porous Metal–Organic Framework Functionalized by a Sulfur-Rich Heterocycle
Source: Nanomaterials (Basel). 2022 Dec 1;12(23):4281. doi: 10.3390/nano12234281 (PMC9737143; doi:10.3390/nano12234281)
Supplement: Supplementary file 1 [file nanomaterials-12-04281-s001.zip › nanomaterials-2034789-supplementary.pdf]

Enhanced Adsorption Selectivity of Carbon Dioxide and Ethane on Porous Metal–Organic Framework Functionalized by a Sulfur-Rich Heterocycle

Vadim A. Dubskikh [1], Konstantin A. Kovalenko [1], Anton S. Nizovtsev [1,2], Anna A. Lysova [1], Denis G. Samsonenko [1], Danil N. Dybtsev [1,*] and Vladimir P. Fedin [1]

[1] Nikolaev Institute of Inorganic Chemistry, Siberian Branch of Russian Academy of Sciences, 3 Acad. Lavrentiev Ave., Novosibirsk 630090, Russia
[2] Novosibirsk State University, 2 Pirogov Street, Novosibirsk 630090, Russia
* Correspondence: dan@niic.nsc.ru

Table S1. Crystal data and structure refinement for **1**.

Parameter	**1**
Empirical formula	$C_{38}H_{39}N_5O_{11}S_4Zn_2$
M, g/mol	1000.72
Crystal system	*Triclinic*
Space group	*P*−1
a, Å	12.6173(2)
b, Å	12.6361(2)
c, Å	14.0187(2)
α, deg.	84.9519(6)
β, deg.	84.8989(7)
γ, deg.	84.4553(7)
V, Å^3	2208.66(6)
Z	2
D(calc.), g/cm^3	1.505
μ, mm^{-1}	1.337
F(000)	1028
Crystal size, mm	0.15 × 0.06 × 0.06
θ range for data collection, deg.	2.28–33.16
Index range	$-19 \le h \le 19$, $-19 \le k \le 18$, $-21 \le l \le 21$
Reflections collected / independent	62013 / 16847
R_{int}	0.0463
Reflections with $I > 2\sigma(I)$	12562
Goodness-of-fit on F^2	1.075
Final *R* indices [$I > 2\sigma(I)$]	$R_1 = 0.0429$, $wR_2 = 0.1231$

R indices (all data)	$R_1 = 0.0602$,<br>$wR_2 = 0.1299$
Largest diff. peak / hole, e/Å^3	0.875 / − 0.735

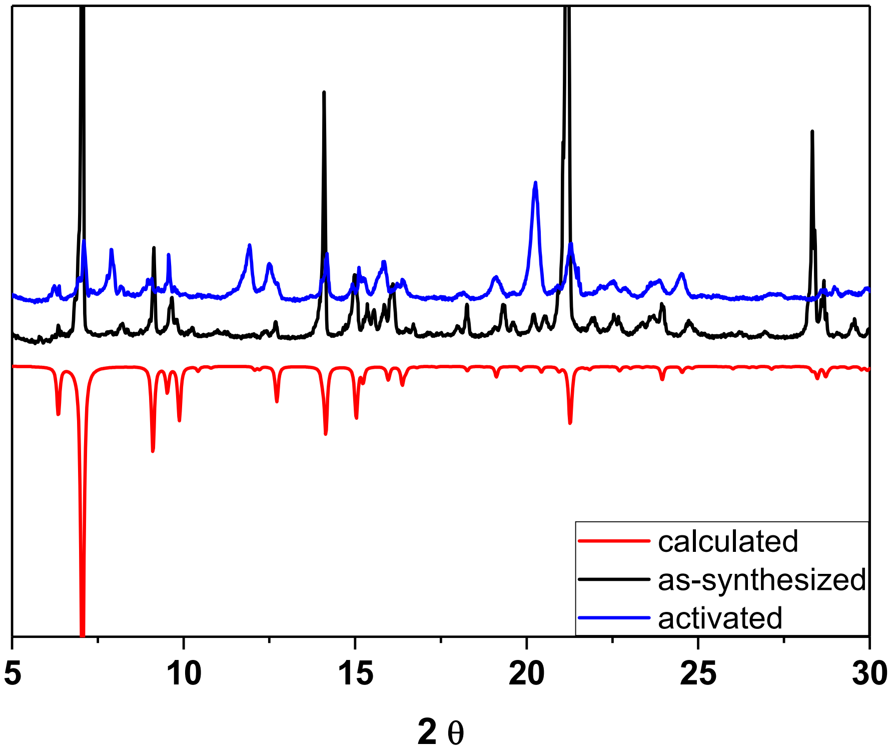

Figure S1. Powder X-ray diffraction patterns of **1** as-synthesized (black), calculated from the single crystal X-ray diffraction data (red) and after activation (blue).

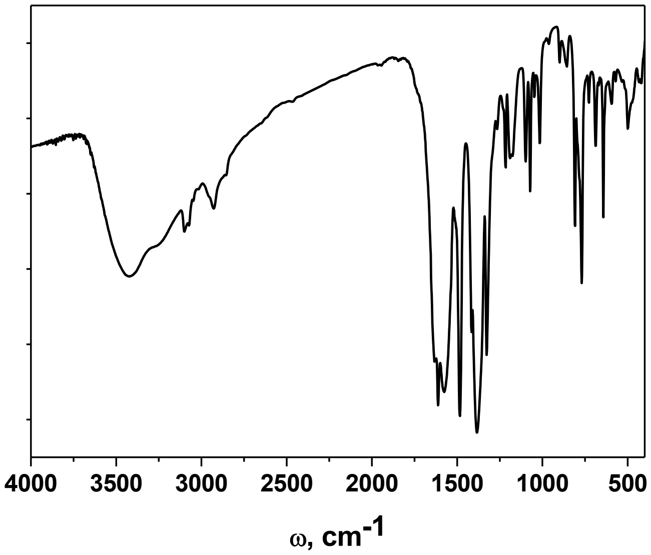

Figure S2. IR spectrum of **1**.

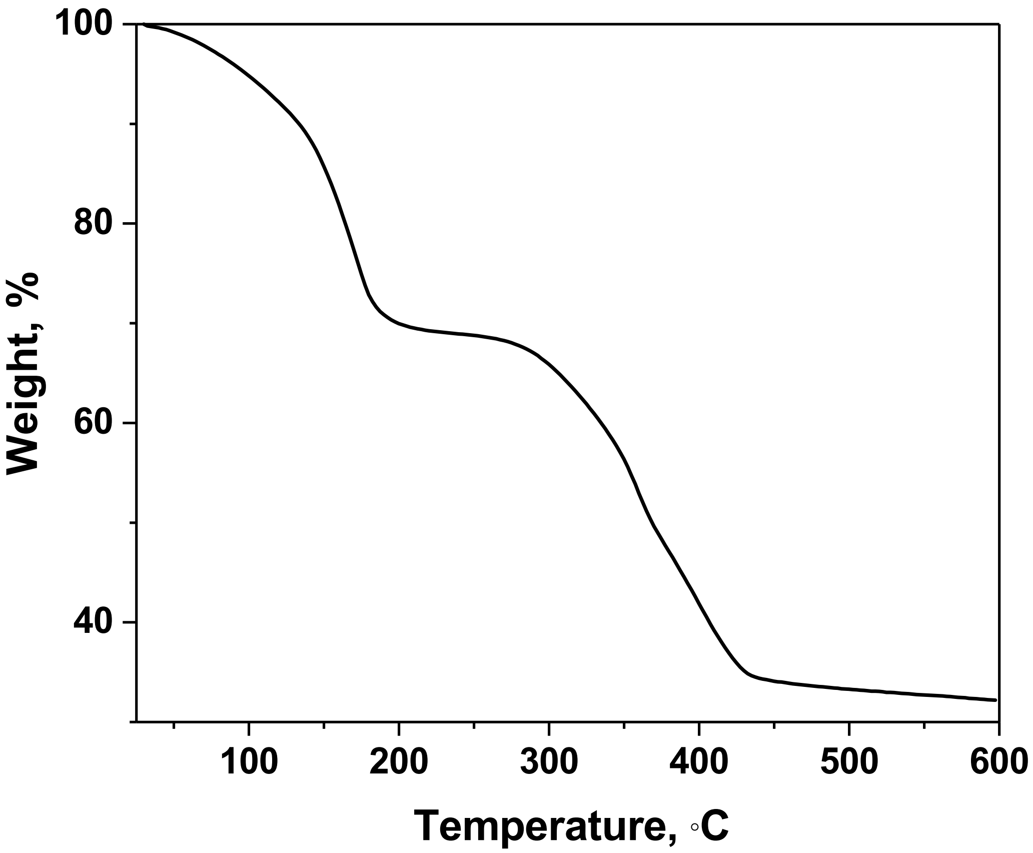

Figure S3. TG curve for **1**.

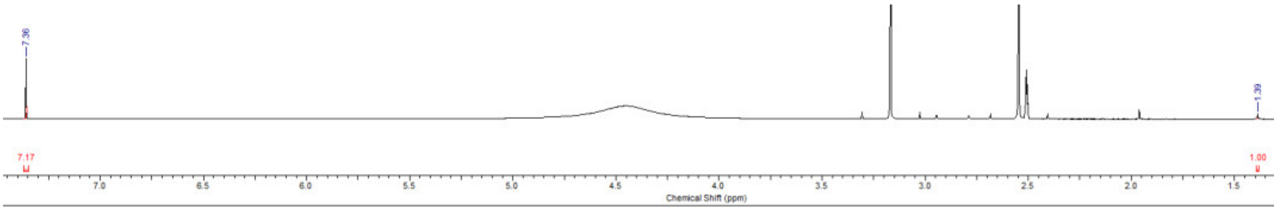

Figure S4. ^{1}H NMR spectrum of d6-dimethyl sulfoxide solution of the digested **1** after adsorption of benzene and cyclohexane from the liquid phase (1:1 v/v).

Table S2. The parameters of porous structure of **1a**.

Specific surface area / $m^2 \cdot g^{-1}$			V_{pore} / $cm^3 \cdot g^{-1}$		$V_{ads}(N_2)^a$/ $cm^3(STP) \cdot g^{-1}$
**Langmuir**	**BET**	**DFT**	**Total**^{a}	**DFT**	
1098	952.0	936.3	0.395	0.369	255

^{a} measured at $P/P_0 = 0.95$.

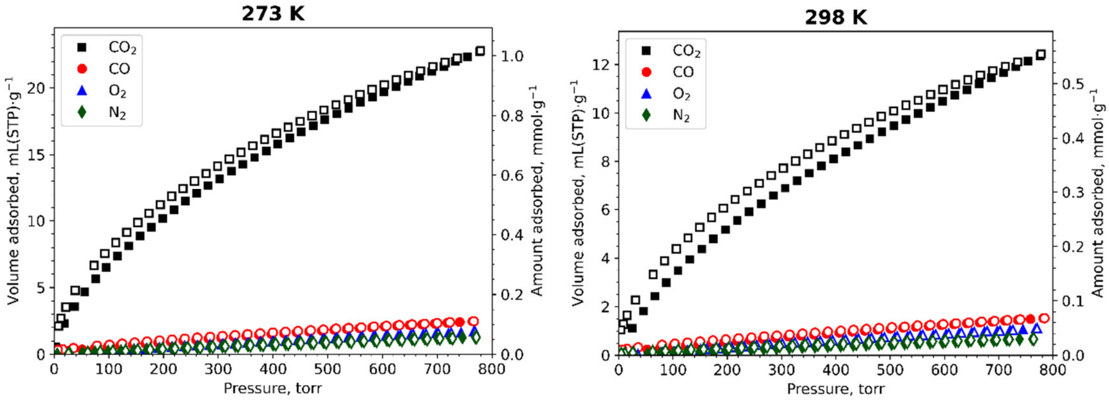

Figure S5. Adsorption and desorption isotherms of N_2, O_2, CO and CO_2 at 273 K (left) and 298 K (right) for **1a**.

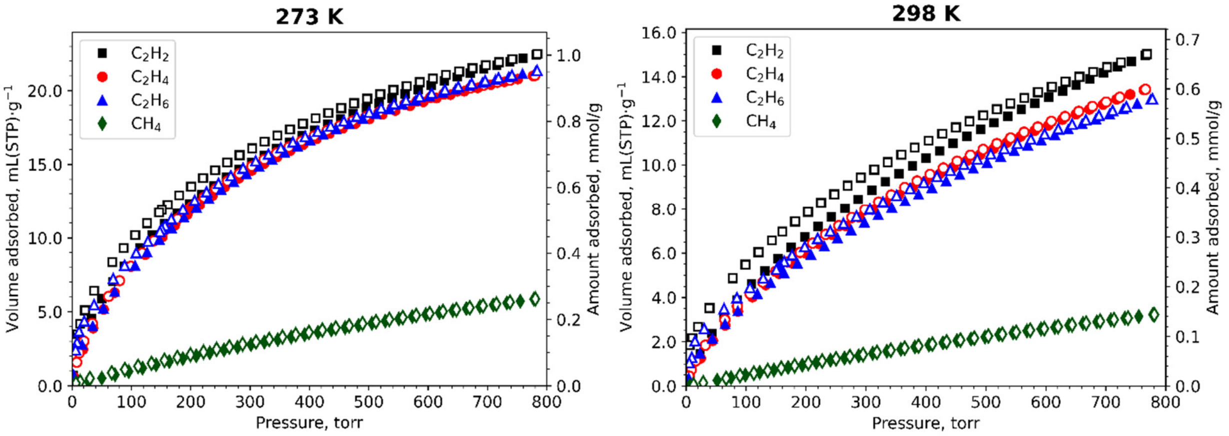

Figure S6. Adsorption and desorption isotherms of CH_4, C_2H_2, C_2H_4 and C_2H_6 at 273 K (left) and 298 K (right) for **1a**.

Heats of adsorption

Isotherms measured at two different temperatures were fitted by virial equation (S1).

$$lnp = lnn + \frac{1}{T}\sum_i A_i \cdot n^i + \sum_j B_j \cdot n^j \qquad \text{(S1)}$$

Values of virial coefficients could be found in Table S3, whereas fit plots are shown in Figure S7.

Table S3. Virial coefficients A_i and B_i for gas adsorption isotherms at 273 K and 298 K on **1a**.

Gas	Coefficients
CO_2	$A_0 = -3776$, $A_1 = 1117$, $A_2 = -1099$, $A_3 = 458.2$, $B_0 = 12.10$
CH_4	$A_0 = -2476$, $A_1 = 345.8$, $B_0 = 10.10$
N_2	$A_0 = -2304$, $B_0 = 11.25$
C_2H_2	$A_0 = -4349$, $A_1 = 2313$, $A_2 = -970.1$, $A_3 = 431.3$, $B_0 = 13.63$, $B_1 = -4.157$
C_2H_4	$A_0 = -3856$, $A_1 = 1058$, $A_2 = -1150$, $A_3 = 688.1$, $B_0 = 12.22$
C_2H_6	$A_0 = -4250$, $A_1 = 1808$, $A_2 = -2439$, $A_3 = 1360$, $B_0 = 13.18$

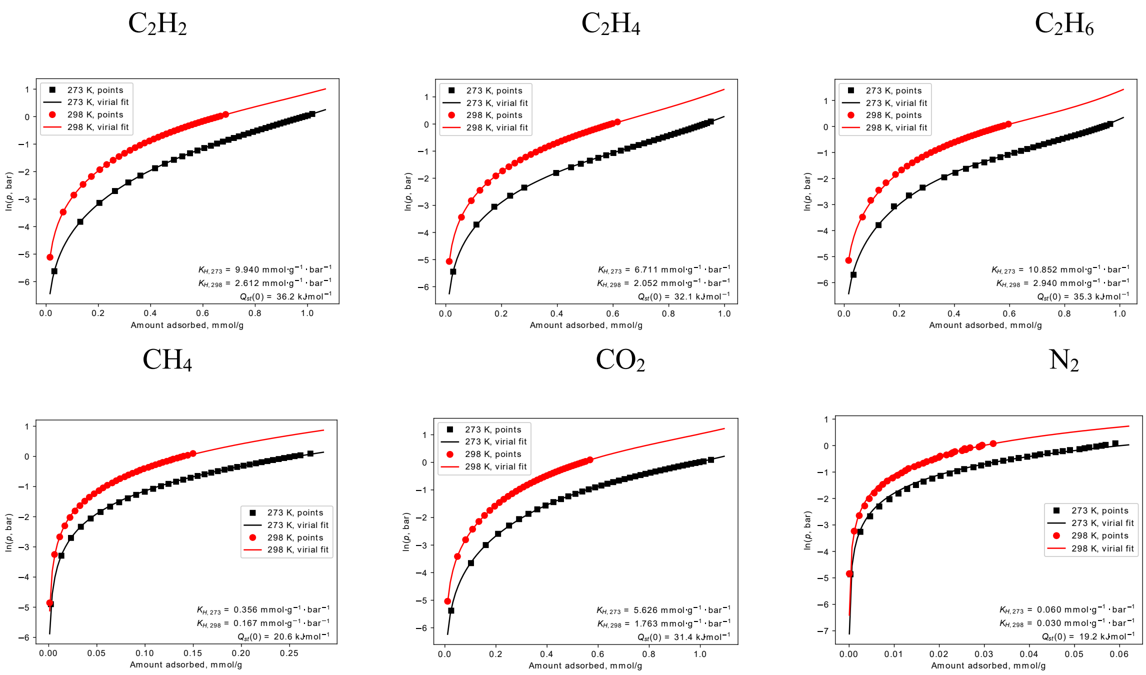

Figure S7. Fits of isotherms by virial equations.

Isosteric heats of adsorption were calculated using equation (S2).

$$\Delta H^{\circ} = R \cdot \sum_{i} A_i \cdot n^i \tag{S2}$$

The corresponding graphs are shown in Figure S8. The zero coverage heats of adsorption are presented in Table S4.

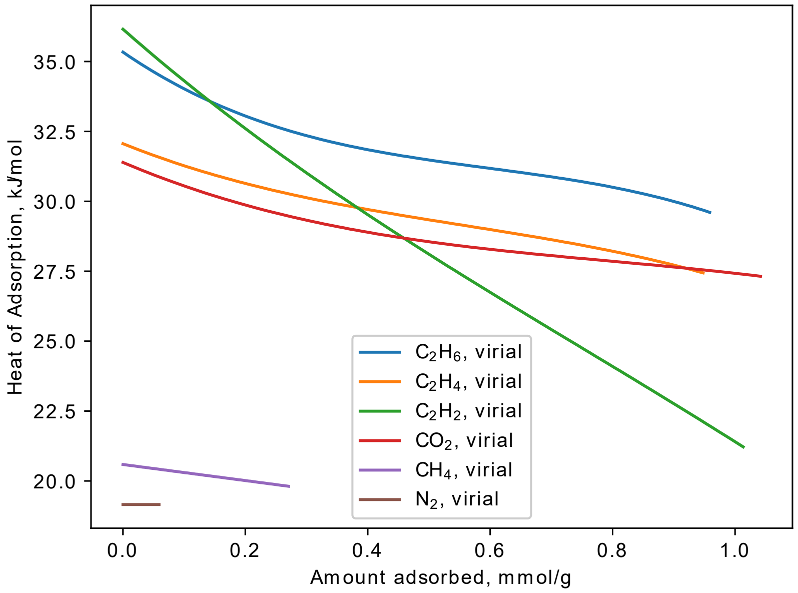

Figure S8. Isosteric heats of adsorption of N_2, CH_4, CO_2, C_2H_2, C_2H_4 and C_2H_6 on **1a** calculated by virial approach as a function of amount adsorbed.

Table S4. Zero coverage heats of adsorption in kJ/mol.

Gas	$Q_{st}(0)$, kJ/mol
CO_2	31.4
CH_4	20.6
N_2	19.2
C_2H_2	36.2
C_2H_4	32.1
C_2H_6	35.3

Henry constants

Henry constants were calculated using virial coefficients by equation (S3):

$$K_h = exp\left[\frac{-A_0}{T} - B_0\right] \tag{S3}$$

Table S1. Henry constants for gas adsorption on **1a** in mmol·g^{-1}·bar^{-1} at 273 K and 298 K obtained by virial approach.

Gas\Temperature	273 K	298 K
CO_2	5.626	1.763
CH_4	0.356	0.167
N_2	0.060	0.030
C_2H_2	9.940	2.612
C_2H_4	6.711	2.052
C_2H_6	10.852	2.940

Gas separation

We have evaluated selectivity factors for separation of binary gas mixtures by three different methods: i) as ratio of amount adsorbed; ii) as ratio of corresponding Henry constants; and iii) by Ideal Adsorbed Solution Theory (IAST) [1] calculations which possess to estimate selectivity factors at different gas mixture compositions and total pressures.

Fit of adsorption isotherms

Adsorption isotherms were fitted by the most appropriate model for IAST calculations. Fittings were performed for isotherms in mL/g–torr units, so parameters are in the corresponding units. Models used and corresponding fitted parameters are summarized in Table S6. Fitted isotherms are shown in Figure S9. Among the models that give close fitting results (R^2, residual sum of squares, parameters errors, good coincidence by eye), preference was given to model with physical basis, and we tried to avoid use the empirical equations (Freundlich) because they are not thermodynamically consistent. So, Freundlich and Langmuir–Freundlich equations have not linear

behavior at low pressures. In case of C_2H_6 adsorption isotherms we were forced to use Langmuir–Freundlich equation as far as all other models did not give satisfactory results of fitting.

Equations used are:

Langmuir model: $n[\text{mL/g}] = \frac{wbp[\text{torr}]}{1+bp[\text{torr}]}$

Dual-Site Langmuir model: $n[\text{mL/g}] = \frac{w_1 b_1 p[\text{torr}]}{1+b_1 p[\text{torr}]} + \frac{w_2 b_2 p[\text{torr}]}{1+b_2 p[\text{torr}]}$

Langmuir-Freundlich model: $n[\text{mL/g}] = \frac{wbp[\text{torr}]^{1/t}}{1+bp[\text{torr}]^{1/t}}$

The consistency of isotherms fitting by appropriate model is also confirmed by comparison of Henry constants calculated both by virial approach and from fitted parameters obtained. By definition, Henry constant is a limit at $p \to 0$ of amount adsorbed function with respect to pressure:

$$K_H = \lim_{p \to 0} \left(\frac{dn(p)}{dp} \right)$$

For multi-site Langmuir equations K_H can be easily calculated:

$$K_H = \lim_{p \to 0} \left(\frac{d \sum_i \left(\frac{w_i b_i p}{1 + b_i p} \right)}{dp} \right) = \sum_i w_i \, b_i$$

Table S6. Fitted parameters for adsorption isotherms on **1a** at 273 K and 298 K and corresponding Henry constants.

**Gas**	**Final set of parameters**		**Henry constant, mmol·g^{-1}·bar^{-1}**	
	**273 K**	**298 K**	**273 K**	**298 K**
CO_2	Langmuir-Freundlich<br>$w = 127.2$, $b = 0.002562$,<br>$t = 1.499$<br>$R^2 = 0.99997$	Langmuir-Freundlich<br>$w = 66.25$, $b = 0.01801$,<br>$t = 1.374$<br>$R^2 = 0.99988$	—	—
CH_4	Langmuir<br>$w = 19.08$, $b = 0.0005665$<br>$R^2 = 0.99997$	Langmuir<br>$w = 15.71$, $b = 0.000331$<br>$R^2 = 0.99995$	0.362	0.174
N_2	Langmuir<br>$w = 6.963$, $b = 0.0002944$<br>$R^2 = 0.99966$	Langmuir<br>$w = 4.726$, $b = 0.0002159$<br>$R^2 = 0.99876$	0.132	0.034
C_2H_2	Dual-Site Langmuir<br>$w_1 = 30.3$, $b_1 = 0.001566$,<br>$w_2 = 5.92$, $b_2 = 0.03427$<br>$R^2 = 0.99998$	Dual-Site Langmuir<br>$w_1 = 38.28$, $b_1 = 0.000517$,<br>$w_2 = 4.52$, $b_2 = 0.01198$<br>$R^2 = 0.99999$	8.376	2.474
C_2H_4	Dual-Site Langmuir<br>$w_1 = 27.8$, $b_1 = 0.002664$,	Dual-Site Langmuir<br>$w_1 = 29.19$, $b_1 = 0.0007506$,	6.670	2.048

	$w_2 = 2.305$, $b_2 = 0.05435$<br>$R^2 = 1.00000$	$w_2 = 3.049$, $b_2 = 0.01289$<br>$R^2 = 1.00000$		
C_2H_6	Langmuir-Freundlich<br>$w = 37.9$, $b = 0.007488$,<br>$t = 1.291$<br>$R^2 = 0.99966$	Langmuir-Freundlich<br>$w = 71.41$, $b = 0.002387$,<br>$t = 1.468$<br>$R^2 = 0.99995$	—	—

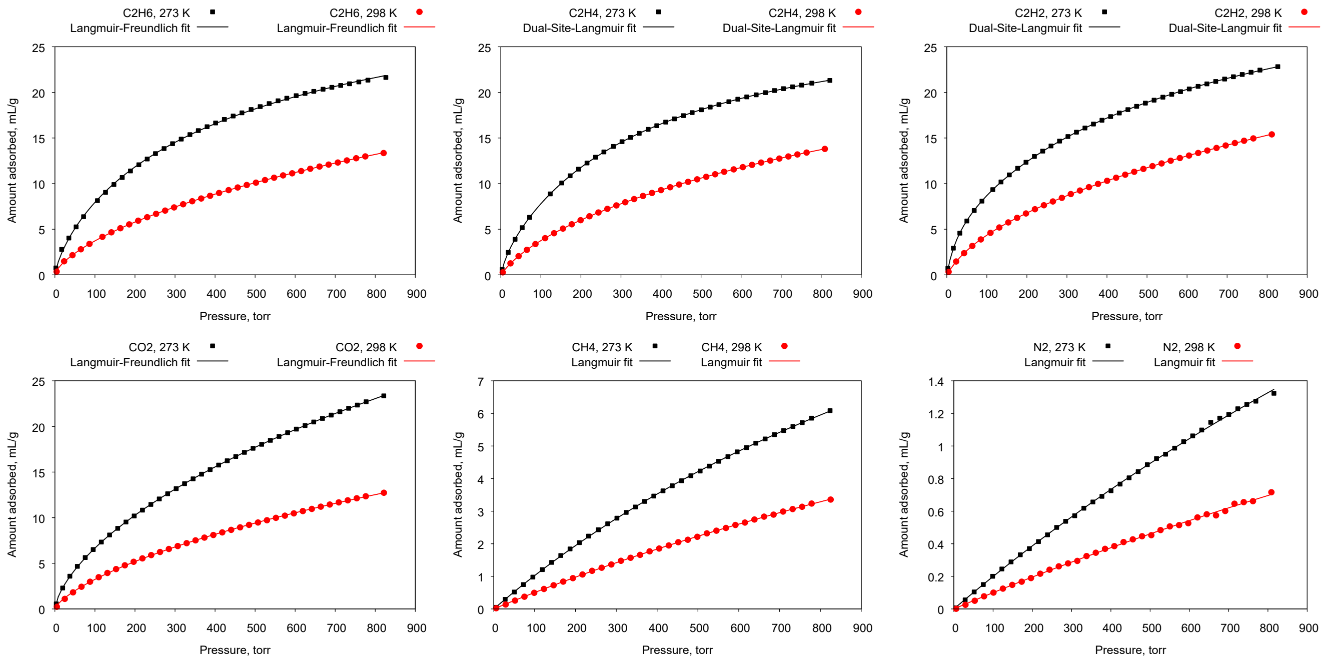

Figure S9. Fits of isotherms by appropriate models.

Selectivity calculations

Selectivity factors were evaluated by three commonly used methods:

(i) As the molar ratio of the adsorption quantities at the relevant partial pressures of the gases:

$$S = \frac{n_1/n_2}{p_1/p_2}, \quad \text{(S2)}$$

where S is the selectivity factor, n_i represents the adsorbed amount of component i, and p_i represents the partial pressure of component i.

(ii) As a ratio of Henry constants which corresponds to the slope of the adsorption isotherm at very low partial pressures:

$$S = \frac{K_{H1}}{K_{H2}} \quad \text{(S3)}$$

(iii) By ideal adsorbed solution theory (IAST). The relationship between P, y_i and x_i (P — the total pressure of the gas phase, y_i — mole fraction of the i-component in gas phase, x_i — mole fraction of the i-component in absorbed state) is defined according to the IAST theory [1]:

$$\int_{p=0}^{p=\frac{Py_1}{x_1}} n_1\,(p)dlnp = \int_{p=0}^{p=\frac{Py_2}{x_2}} n_2\,(p)dlnp \tag{S4}$$

In this case the selectivity factors were determined as:

$$S = \frac{y_2 x_2}{y_1 x_1} = \frac{x_1(1 - y_1)}{y_1(1 - x_1)} \tag{S5}$$

The results are summarized in Table 2 and in Figures S10, S11.

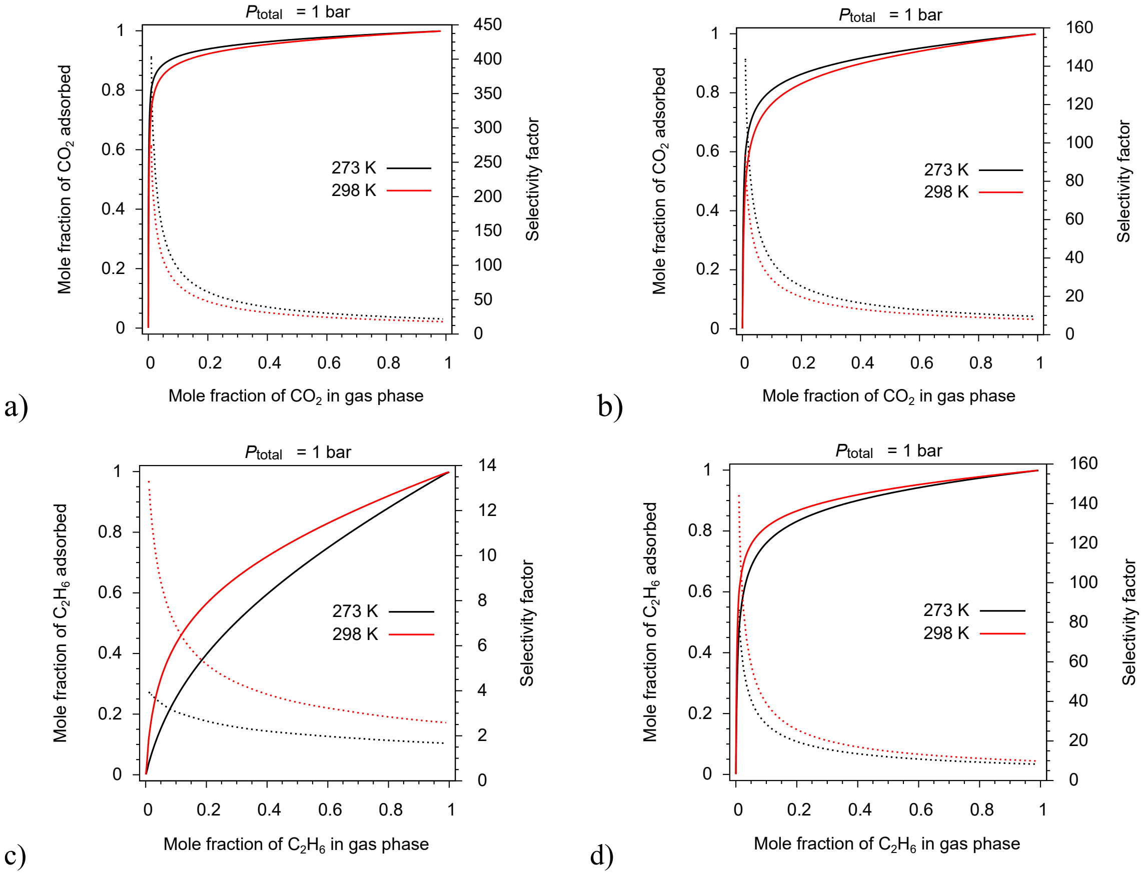

Figure S10. Prediction of adsorption equilibrium by IAST (solid lines) and dependence of selectivity factors on gas phase composition (dashed lines) for binary gas mixtures (total pressure 1 bar): a) CO_2/N_2; b) CO_2/CH_4; c) C_2H_6/C_2H_2; d) C_2H_6/CH_4.

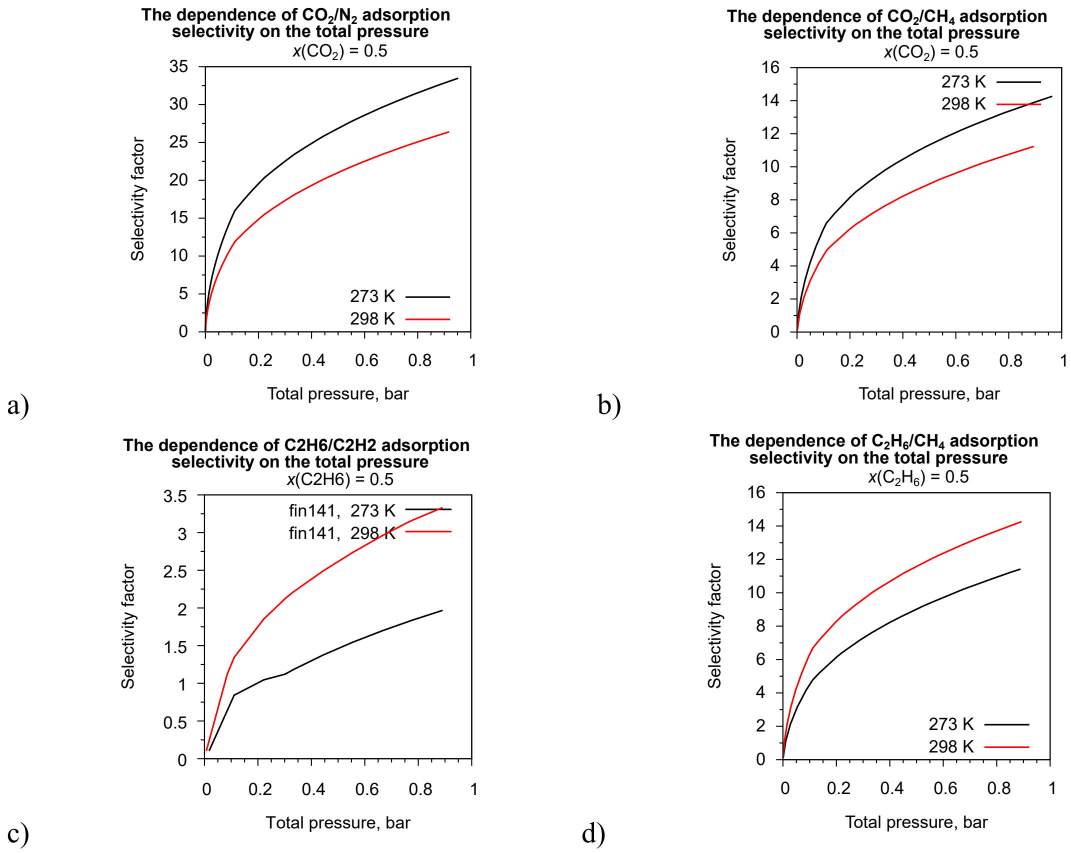

Figure S11. Dependence of selectivity factors on total gas pressure for equimolar binary gas mixtures: a) CO_2/N_2; b) CO_2/CH_4; c) C_2H_6/C_2H_2; d) C_2H_6/CH_4.

Table S7. The parameters of porous structure of [$Zn_2(bdc)_2$(dabco)], [$Zn_2(tdc)_2$(dabco)] [2] and [$Zn_2(ttdc)_2$(bpy)] (**1a**, this work).

MOF	Crystal density / $cm^3 \cdot g^{-1}$	$V_{pore}{}^a$ / $cm^3 \cdot g^{-1}$	$V_{pore}{}^a$ / $cm^3 \cdot cm^{-3}$	S_{BET} / $m^2 \cdot g^{-1}$	S_{BET} / $m^2 \cdot cm^{-3}$
**[$Zn_2(bdc)_2$(dabco)]**	0.83	0.75	0.62	1450	1204
**[$Zn_2(tdc)_2$(dabco)]**	0.94	0.68	0.64	1553	1460
**[$Zn_2(ttdc)_2$(bpy)]**	1.11	0.40	0.44	952	1057

[a] measured at $P/P_0 = 0.95$.

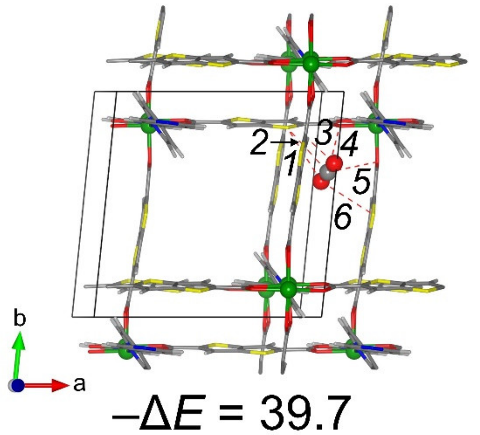

–Δ*E* = 39.7

Site I.A	CO$_2$@MOF		
Contact	*r*, Å	q_{guest}, |e|	q_{host}, |e|
1 **(O···S)**	3.53	-0.15	0.13
2 **(C···S)**	3.55	0.27	0.13
3 **(O···H)**	3.18	-0.15	0.05
4 **(C···O)**	3.02	0.27	-0.23
5 **(C···O)**	3.16	0.27	-0.24
6 **(O···S)**	3.46	-0.15	0.12

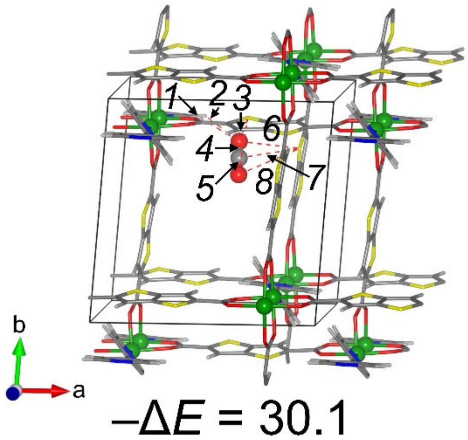

–Δ*E* = 30.1

Site I.B	CO$_2$@MOF		
Contact	*r*, Å	q_{guest}, |e|	q_{host}, |e|
1 **(O···H)**	2.73	-0.13	0.06
2 **(O···H)**	2.80	-0.13	0.04
3 **(O···S)**	3.79	-0.13	0.14
4 **(C···S)**	3.59	0.28	0.14
5 **(O···S)**	3.79	-0.14	0.14
6 **(O···S)**	3.57	-0.13	0.14
7 **(C···S)**	3.42	0.28	0.14
8 **(O···S)**	3.68	-0.14	0.14

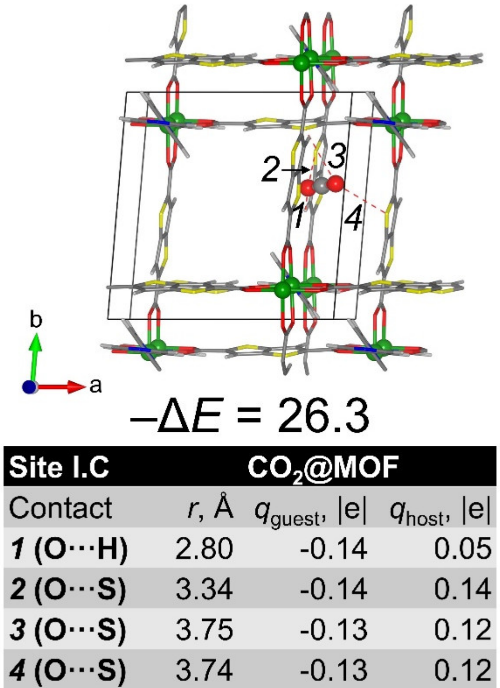

–Δ*E* = 26.3

Site I.C	CO$_2$@MOF		
Contact	*r*, Å	q_{guest}, |e|	q_{host}, |e|
1 **(O···H)**	2.80	-0.14	0.05
2 **(O···S)**	3.34	-0.14	0.14
3 **(O···S)**	3.75	-0.13	0.12
4 **(O···S)**	3.74	-0.13	0.12

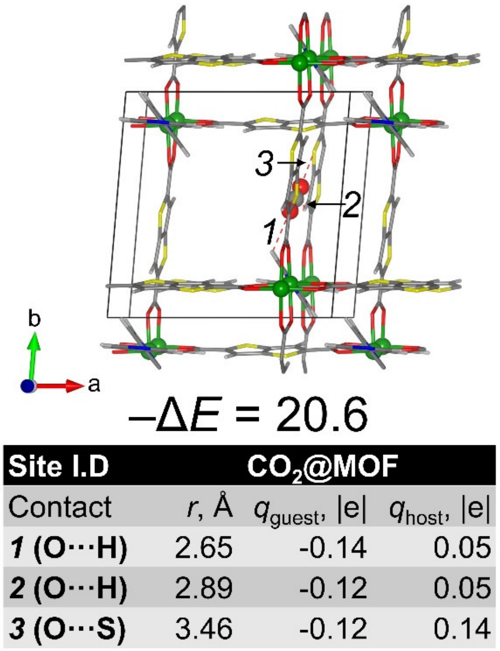

–Δ*E* = 20.6

Site I.D	CO$_2$@MOF		
Contact	*r*, Å	q_{guest}, |e|	q_{host}, |e|
1 **(O···H)**	2.65	-0.14	0.05
2 **(O···H)**	2.89	-0.12	0.05
3 **(O···S)**	3.46	-0.12	0.14

Figure S12. Calculated interatomic distances (Å) of the shortest $X_{guest}\cdots Y_{host}$ contacts (red dashed lines) and the corresponding Hirshfeld partial charges for CO_2 molecule adsorbed at the most relevant sites I.A, I.B, I.C, and I.D inside [$Zn_2(ttdc)_2(bpy)$] pores. Adsorption energies (Δ*E*) are given in $kJ\cdot mol^{-1}$. Color code: Zn (green), S (yellow), O (red), N (blue), C (gray), H (light gray).

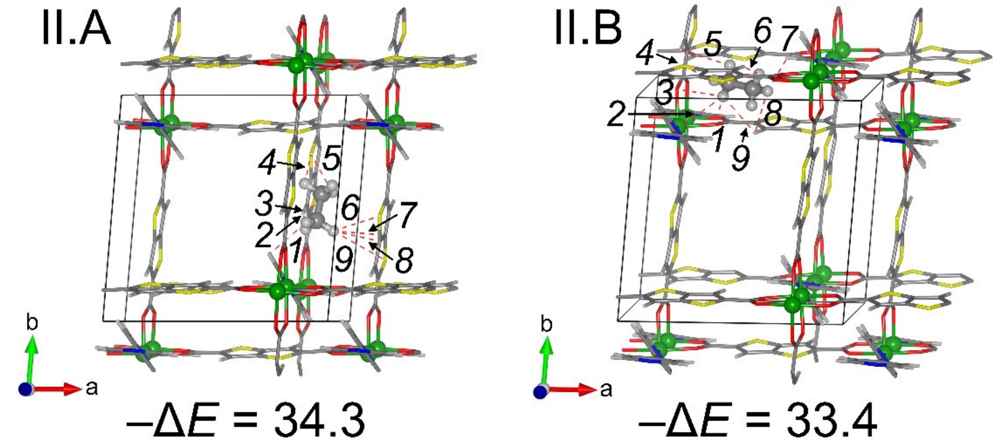

Site II.A	C_2H_6@MOF		
Contact	*r*, Å	q_{guest}, |e|	q_{host}, |e|
1 **(H···H)**	2.89	0.03	0.06
2 **(H···H)**	2.73	0.03	0.06
3 **(H···H)**	2.89	0.03	0.06
4 **(H···S)**	3.48	0.03	0.15
5 **(H···S)**	3.30	0.03	0.15
6 **(H···S)**	3.72	0.02	0.12
7 **(H···C)**	2.90	0.02	-0.04
8 **(H···C)**	3.06	0.02	-0.04
9 **(H···S)**	3.41	0.02	0.12

Site II.B	C_2H_6@MOF		
Contact	*r*, Å	q_{guest}, |e|	q_{host}, |e|
1 **(H···O)**	2.65	0.03	-0.22
2 **(H···N)**	3.13	0.03	-0.07
3 **(H···O)**	3.24	0.03	-0.22
4 **(H···S)**	3.36	0.02	0.13
5 **(H···H)**	2.90	0.03	0.06
6 **(H···H)**	2.46	0.03	0.05
7 **(H···H)**	2.75	0.03	0.06
8 **(H···H)**	2.77	0.03	0.06
9 **(H···H)**	2.77	0.03	0.06

Figure S13. Calculated interatomic distances (Å) of the shortest $X_{guest}\cdots Y_{host}$ contacts (red dashed lines) and the corresponding Hirshfeld partial charges for C_2H_6 molecule adsorbed at the most relevant sites II.A and II.B inside $[Zn_2(ttdc)_2(bpy)]$ pores. Adsorption energies (ΔE) are given in kJ·mol^{-1}. Color code: Zn (green), S (yellow), O (red), N (blue), C (gray), H (light gray).

References:

1. Myers, A.L.; Prausnitz, J.M. Thermodynamics of mixed-gas adsorption, *AIChE J.* **1965**, 11, 121-127.

2. Bolotov, V.A.; Kovalenko, K.A.; Samsonenko, D.G.; Han, X.; Zhang, X.; Smith, G.L. McCormick, L.J.; Teat, S.J.; Yang, S.; Lennox, M.J.; Henley, A.; Besley, E.; Fedin, V.P.; Dybtsev, D.N.; Schröder, M. Enhancement of CO_2 uptake and selectivity in a metal−organic framework by the incorporation of thiophene functionality. *Inorg. Chem. 2018*, 57, 5074−5082.
